# Critical Consciousness for Connectivity: Decoding Social Isolation Experienced by Latinx and LGBTQ+ Youth Using a Multi-Stakeholder Approach to Health Equity

**DOI:** 10.3390/ijerph191711080

**Published:** 2022-09-04

**Authors:** Nancy Vargas, Jesse L. Clark, Ivan A. Estrada, Cynthia De La Torre, Nili Yosha, Mario Magaña Alvarez, Richard G. Parker, Jonathan Garcia

**Affiliations:** 1 College of Public Health and Human Sciences, Oregon State University, Corvallis, OR 97331, USA; 2 David Geffen School of Medicine, University of California Los Angeles, Los Angeles, CA 90095, USA; 3 Benton County Public Health, Corvallis, OR 97330, USA; 4 Outside the Frame, Portland, OR 97209, USA; 5 Associação Brasileira Interdisciplinar de AIDS, Rio de Janeiro 20071-907, Brazil; 6 Hallie E. Ford Center for Healthy Children and Families, College of Public Health and Human Sciences, Oregon State University, Corvallis, OR 97331, USA

**Keywords:** Latinx LGBTQ+ youth, social isolation, critical consciousness, solidarity, community-engaged qualitative research

## Abstract

Systemic oppression creates a context in which Latinx LGBTQ+ youth experience social isolation. Social isolation has been associated with mental and physical health disparities, including disproportionate levels of depression, substance use, self-harm, and attempted suicide. These disparities are often magnified in rural and suburban areas with fewer identity-affirming spaces. This community-engaged study reports on the formative process of developing a Latinx LGBTQ+ telenovela (soap opera) allyship intervention based on critical consciousness theory. We conducted eight focus groups with community advisory boards, which included Latinx LGBTQ+ youth (*n* = 12), health and social service providers serving LGBTQ+ youth (*n* = 10), 4-H Latinx alumni youth (*n* = 12), and 4-H Latinx parents (*n* = 8). We interviewed nine Latinx LGBTQ+ youth enrolled in a film-making workshop. As a result of our multi-stakeholder approach, we: (1) described how stakeholders reflected on and decoded intersectional isolation on the individual, community, and structural levels; and (2) identified ways that stakeholders suggested taking action by improving access to resources to address social isolation, provide culturally competent healthcare, and co-create an enabling social environment. Our study indicated the importance of tapping into core values and intersectional identities to build solidarity among and within marginalized groups to dismantle oppressive systems.

## 1. Background

Oppressive systems, institutions, and cultural norms may lead LGBTQ+ youth to experience social isolation, which represents a “lack of a sense of belonging, the inability to engage and connect with others, and the neglect or deterioration of social relationships” [1]. Families, communities, and institutions can socialize youth into isolation by promoting toxic masculinity and strategic invisibility as coping strategies for youth who do not conform to hetero- and cisgender normativity [2,3,4,5,6]. Nonconforming youth often learn to negate the parts of their identities that they perceive as undesirable depending on their social setting—reinforcing systems of oppression by attempting to “pass” or fit in [5,7]. These experiences with isolation, rejection, and self-denial during critical developmental phases may shape youths’ sense of self and self-worth, having lasting effects on their mental health and behavioral skills across the life course [8,9,10].

Latinx LGBTQ+ youth experience intersectional marginalization and stigma as ethnic, sexual, and gender minorities [4,11], which may result in heightened social isolation [1]. For Latinx youth, their families, religious organizations, and ethnic and cultural enclaves are key sources of social, spiritual, and material support [12,13], and can be protective against the consequences of racism and xenophobia [12,14,15], especially in predominantly White contexts, such as Oregon [14,16]. However, *familismo*—a construct that describes how Latinx cultures value the family as a major source of social support and that places respect for the family over the individual—may also be a major driver of isolation by causing Latinx youth to fear disclosing their LGBTQ+ identities and avoid disrespecting the family [17,18]. LGBTQ+ youth may also lose access to essential material resources, such as housing security, as a consequence of coming out and being rejected by their families and communities [19,20]. 

LGBTQ+ youth of color are more prone to experience rejection and bullying from peers in schools [21,22,23]; this bullying has been associated with attempted suicide [24]. Recent studies document a lack of intersectional safe spaces for LGBTQ+ youth of color, which may be worse in rural places. A study conducted by GLSEN in 2020 found that 54.9% of Latinx LGBTQ+ students felt unsafe in schools due to their sexual orientation, 44.2% felt unsafe in relation to their gender expression, and 22.3% felt unsafe in relation to their race or ethnicity [11]. According to their report, “Latinx LGBTQ students in majority-White schools were more likely to have a GSA [Genders and Sexualities Alliances, also known as Gay-Straight Alliances] than those in majority-Latinx schools” (pg. xviii). They found that 22.5% of Latinx LGBTQ+ youth “were taught positive representations of LGBTQ people, history, or events” [11] (pg. xix). Those who had a curriculum in school with positive LGBTQ+ inclusion were less likely to feel unsafe because of their sexual orientation and gender expression and felt more connected to their school community.

Various aspects of social isolation among LGBTQ+ youth have been associated with higher rates of self-harm [25,26], suicidal ideation and attempts [27,28,29], depression [30,31,32], substance use [31,33,34,35], and inequitable access to health services [36] compared to their cisgender/heterosexual counterparts. Findings from the Oregon Health Authorities’ 2019 Healthy Teen Survey showed that among 11th graders who identified as non-binary and gender non-conforming, 54.2% reported feeling sad or hopeless for at least two weeks, 37.2% had seriously considered suicide within the last year, and 13.8% had attempted suicide within the last 12 months [37]. Of 655 non-binary and gender non-conforming youth, 40.6% felt their mental health professional, counselor, or social worker did not meet their emotional or mental health needs [38]. This failure to meet their psychosocial needs results from and drives social isolation, which requires altering the social environment through peer-based efforts to facilitate access to quality, culturally appropriate mental health services.

Interventions to change the social environment to address isolation may foster positive identity development [1]. Supportive relationships and engagement in identity-safe environments result in resilience among LGBTQ+ youth of color [22] and attenuate the health consequences resulting from social isolation [1]. Providing alternate or supplemental social support systems and peer networks can be particularly important in buffering the social isolation experienced in rural areas and suburban towns [39,40]. This issue is particularly pertinent in Oregon [41], where many Latinx families are migrant farmworkers living in geographically isolated areas. 

In this context, positive youth development programs, such as the Oregon 4-H Outreach Youth Development Education program, have been committed to the wellbeing of underserved populations, including children, youth, and adults from all different racial, ethnic, and cultural backgrounds [42]. The 4-H Outreach Leadership Institutes have a history of bringing together Latinx children and young adults from throughout the state to provide enrichment opportunities, foster civic responsibility, and to expand young people’s educational opportunities. However, programming that trains youth as effective allies for their LGBTQ+ peers is needed within 4-H to become more meaningfully inclusive. While allyship-based programs, such as GSAs, have been useful in addressing social isolation in formal school contexts, few of these programs focused on addressing the intersectional marginalization suffered by Latinx and other LGBTQ+ youth of color [1]. 

A solidarity-based approach is critically necessary to fill this gap by engaging multiple stakeholders (i.e., LGBTQ+ Latinx youth, Latinx peers, Latinx parents, and health and social service providers) to counterbalance politically driven, identity-based conflicts in the USA, which generate tensions among different marginalized groups and, in turn, reproduce oppressive social norms [43,44]. This study reports on in-depth focus group discussions with key stakeholders responsible for cultivating an enabling environment that can address social isolation and oppression among LGBTQ+ Latinx youth in 4-H. Our approach is based on a theoretical framework that guides decoding social isolation to confront oppression and generate empathy and solidarity with youth who are marginalized because of their ethnicity, place of residence, history of migration, and socioeconomic status, as well as their sexuality, gender identity, and expression. 

## 2. Theoretical Foundation: Critical Consciousness for Solidarity

We draw on critical consciousness theory to guide the development of a popular education intervention that engages youth in critical reflection, discussion, and action [45,46,47,48,49]. The Brazilian educator, Paulo Freire, argued that to achieve critical consciousness, the oppressed and their oppressors both need to grow in awareness of how they interact with the social world, work together to build community, and commit to change social conditions [46]. Critical reflection on oppressive systems, which were largely class-based in Freire’s original approach, identified targets for systemic, community, and individual behavior change. According to Freire, it is first necessary for the oppressed to see the ways in which these systems push them to strive to become the oppressors, or reinforce the oppressive system, and consequently remain willing to dehumanize and cause harm to other oppressed people. Liberating all groups can only be done through praxis—“reflection and action directed at the structures to be transformed” [46] (p. 126). Without this critical reflection and action, systems of oppression will remain internalized and reproduce themselves among the oppressed, perpetuating themselves in daily life by governing social relationships. 

This model encourages participants to challenge “banking models” of education in which elites unidirectionally deposit what is right/wrong and true/false. Bi-directional learning challenges a status quo that further marginalizes oppressed people. Freire advocates for a problem-focused pedagogy in which students co-create new knowledge that directly relates to their lives and is rooted in the hope of changing social conditions. In this process, participants “decode” social oppression by identifying it in their daily lived experience, language, symbols, and cultural framings with the goal of producing transformative change in their understanding of systems of oppression, behavioral strategies, and interpersonal relationships [47,50]. This approach can also be used to understand how relationships of oppression are characterized by the unequal power dynamics that are legitimized by the culture of various institutional spaces (schools, religious organizations, and sports clubs), in peer networks, in families, and within communities.

*Authentic*, courageous dialogue is necessary to raise critical consciousness, in which those playing the role of oppressed/oppressors believe in each other’s ability to be transformed, talk and listen to critically reflect on their realities, and find common ground in their experiences—all with the goal of liberation and social action. Freire describes “cultural inauthenticity” as an instrument of “cultural invasion”:

*For cultural invasion to succeed, it is essential that those invaded become convinced of their intrinsic inferiority. Since everything has its opposite, if those who are invaded consider themselves inferior, they must necessarily recognize the superiority of the invaders. The values of the latter thereby become the pattern for the former. The more invasion is accentuated and those invaded are alienated from the spirit of their own culture and from themselves, the more the latter want to be like the invaders: to walk like them, dress like them, talk like them*.

[46] (p. 153)

In this way, systems of oppression (e.g., White Supremacy, homophobia, transphobia, and xenophobia) divide and conquer marginalized factions by pitting them against each other with the false promise of belonging and gaining the privilege of the oppressor (e.g., “passing”). Authentic dialogue is a way to openly acknowledge power imbalances that are socially corrosive. Rather than reinforcing dichotomies, such as normal/deviant, a culture of authenticity is necessary to develop awareness, pride, and celebration of the self for those who endure systemic oppression. Generating authentic dialogue counter-balances the “culture of silence” and shame and guilt, which drive youth into isolation [46] (p. 33). 

It is important to emphasize that the roles of oppressed and oppressor are fluid and situational. A culture of authenticity also calls for the recognition that in intersectional situations (i.e., where multiple marginalized identities are at play), the oppressed can play the role of oppressors. In this paper, we explore this phenomenon in the ways that LGBTQ+ Latinx youth can be oppressed within Latinx communities that experience oppression. We advance the theory of critical consciousness by showing that the relationship between oppressed and oppressor is not static or binary. We argue that systems of oppression are difficult to dismantle precisely because they involve a complex pattern of intersectional oppression and privilege. That is to say, because of the way systems of oppression shift to perpetuate themselves, they may result in someone who experiences a great degree of oppression perpetuating the system (e.g., a Latino child from a farmworker family in rural Oregon playing the oppressor among his LGBTQ+ peers, who may even come from a higher SES). These shifting and provisional relationships of power and inequity based on axes of class, race, identity, gender, sexuality, and language must be recognized and addressed in order to begin to dismantle the systems of oppressor and oppressed that govern contemporary society.

Whereas previous research describes solidarity as leading to “action taken by the advantaged group for the disadvantaged group [51] (p. 292)”, our study shows that the roles of the advantaged/disadvantaged are situational. We want to move beyond a definition of allyship that has emphasized the essential power dynamic between altruistic helpers who “relinquish social privileges conferred by their group status” to assist those in need [52] (p. 713). Although solidarity may sometimes be driven by “moral beliefs and a resulting imperative to respond” [51] (p. 292), we suggest that from mutual identification (i.e., seeing parts of oneself in another, even though the other parts are different) can emerge a shared interest in dismantling the system of oppression that affects everyone. This type of solidarity is needed to achieve a system of liberation in which celebrating all aspects of youth’s intersectional identities is beneficial to everyone.

This article reports on the formative process of developing an intervention based on critical consciousness theory to identify cultural blind spots, develop skills for dialogue and communication, increase awareness and understanding of the effects of systems of oppression, and ultimately build mutual understanding and solidarity among various marginalized groups. To build solidarity among marginalized groups, we convened community advisory boards (CABs) with multiple stakeholders. First, we sought to identify how Latinx LGBTQ+ youth experience social isolation, and how providers witness the results of isolation on access to health and other social services. Second, in focus groups with two non-LGBTQ+ groups of Latinx stakeholders (4-H Latinx youth and Latinx 4-H parents), we set out to identify implicit biases that could be decoded using critical consciousness and assess the need for enabling social environments in 4-H to foster positive youth development.

## 3. Methods

Our popular education model to transform oppressive systems originated in Brazil [53,54,55]. Our approach was inspired by work conducted in Rio de Janeiro at the Brazilian Interdisciplinary AIDS Association, which trains youth to navigate social relationships and address stigma, prejudice, and discrimination within families, local communities, religious organizations, and school/other educational environments [56,57]. Community-engaged, collaborative research recognizes the knowledge of community stakeholders, promotes engagement in various stages of the research and dissemination, and is driven by the value of social justice and social action [53,54,55]. 

From February 2019 through June 2020, we conducted a community-engaged study with multiple groups of stakeholders to (1) describe the factors they perceived to generate social isolation and those they perceived would prevent or reduce social isolation among LGBTQ+ Latinx youth, (2) identify personal experiences of social isolation, and (3) engage the community to critically reflect on data, analyze social isolation as a result of systems of oppression, and take action by co-creating the intervention toolkit. We conducted 8 focus groups with community advisory boards (CABs), which included Latinx LGBTQ+ youth (*n* = 12), health and social service providers serving LGBTQ+ youth (*n* = 10), 4-H Latinx alumni youth (*n* = 12), and 4-H Latinx parents (*n* = 8). We engaged the four CABs with the overall aim of developing a critical consciousness intervention in the 4-H Outreach Leadership Institute to train 4-H youth as allies for their LGBTQ+ peers. Themes from the focus groups inspired 10 LGBTQ+ Latinx youth to co-create 5 telenovela (soap opera) short films based on their lived experience with social isolation to be used in the intervention toolkit. These methods are summarized in Table 1.

### 3.1. Participant Recruitment

CAB members were recruited based on the following eligibility criteria: (1) In the Latinx LGBTQ+ youth group, participants identified as Latinx or Multi-racial Latinx, were 18–24 years old, and identified as LGBTQ+; 15 LGBTQ+ youth participated in completing the demographics worksheet and notecards exercise, but three of these participants were not included in these analyses because they did not identify as Latinx; (2) 4-H Alumni youth identified as Latinx or Multi-racial Latinx, were 18–24 years old, and participated in 4-H activities during high school; (3) 4-H Parents identified as Latinx or Multi-racial Latinx, were 18 years and older, and had a child who currently or previously participated in 4-H; (4) Health and social service providers were 18 years and older, worked at a community health clinic, youth service organization, outreach program, or other organization that provided direct services to LGBTQ+ Latinx youth. The youth who co-created the videos for the intervention toolkit identified as Latinx or Multi-racial Latinx were 18–24 years old and identified as LGBTQ+. All CAB participants agreed to be audio-recorded. 

Focus groups were recruited from and held in two distinct geographic locations in order to include a variety of participants. For focus groups held in Corvallis, we recruited by first contacting key community-based organizations serving LGBTQ+ youth (e.g., Pride Center, the Women and Gender Equity Center, Centro Cultural Cesar Chavez, and the SOL: LGBTQ+ Multicultural Support Network, which focuses on Queer and Trans People of Color-QTPOC). We asked them to share our recruitment materials on their social media networks and listservs. Recruitment was also conducted at two different drag shows organized by local community organizations. At the first drag show, recruitment was conducted through our existing contacts and chain referrals. We conducted formal tabling at the second drag show to systematically capture a wider audience. The flyers were posted on social media including Snapchat, Twitter, and Instagram. 

Also in Corvallis, 4-H Latinx parents and young alumni were recruited through social media and email contacts the by program coordinator (NV) and Oregon State University’s extension specialist (MMA) who runs the Outreach Leadership Institute, Migrant Summer Camps, and other 4-H programming for Latinx communities throughout Oregon. 

For the focus groups conducted in Portland, Outside the Frame, a leading community-based organization focused on co-creating films with marginalized and homeless youth, and our partner in this project (NY), led recruitment by emailing other community centers and institutions that worked with Latinx LGBTQ+ youth as well as by distributing the flyers at local LGBTQ+ or Latinx events. In addition, they identified Latinx LGBTQ+ participants in their programs fitting the recruitment criteria. Outside the Frame also helped with recruitment for the LGBTQ+ service providers by directly emailing and calling their existing contacts. 

### 3.2. Procedures

#### 3.2.1. Focus Groups with CAB Members

Before each 2 h focus group began, participants were read the verbal consent script and were given space to ask questions. All participants who showed up for the focus groups verbally consented to participate. In a folder, each participant received a name tag, a demographics worksheet, 20 notecards, and an envelope. All were asked to write their first name and their gender pronouns on the name tag. The next step was to complete a demographics worksheet to contextualize each focus group. In this worksheet, LGBTQ+ youth were asked about their age, self-identified race/ethnicity, sexual orientation and gender identity, occupation, and religious affiliation. Health and social service providers were asked their age, occupation, gender identity, and religious affiliation. 4-H Latinx youth and parents were asked their age, gender identity, religious affiliation, and time and role in 4-H. On the same worksheet, participants were asked to recount scenarios that could be used for the telenovela videos (e.g., Describe a scene in which you witnessed or experienced bullying; Describe a scene in which a Latinx LGBTQ+ young person encountered challenges to accessing health and social services), including the situation, context, who was involved, and if/how the situation was resolved. 

Next, they were asked to use their 20 notecards to try to identify 10 factors they believed led to social isolation among Latinx LGBTQ+ youth and 10 factors they believed would protect Latinx LGBTQ+ youth from social isolation. After that activity, they read the factors out loud, the facilitator wrote them on the board, and participants each selected their top three barriers and top three facilitators by using color-coded post-it notes. After this exercise, the initial discussion focused on exploring stories based on those top 3 factors. 

After the first discussion, questions for the corresponding CAB theme were asked, as well as, probing questions or emerging areas that researchers determined required further exploration. This discussion varied by CAB type. LGBTQ+ youth and 4-H youth focused on experiences with bullying and the resources needed to address intersectional marginalization. Parents focused on intersectional marginalization and the meaning of citizenship and community building to address social isolation. Health and social service providers focused on the variety of resources necessary to address social isolation, including daily institutional practices in their workplace. In the last portion of the focus groups, participants broke out into small groups of 2–3 and were instructed to discuss the scenarios they had developed to inform the telenovela videos. At the end of the focus group, they were given information for the film making/telenovela workshop in the summer. 

#### 3.2.2. Interviews with Latinx LGBTQ+ Youth Making Telenovela Films

Following the focus group discussions, we recruited 10 Latinx LGBTQ+ youth to make telenovela-inspired videos about their life experiences with social isolation. Nine out of 10 Latinx LGBTQ+ youth (ages 18–24) who agreed to participate in the filmmaking workshop consented to allow their interviews to be analyzed as data in this research and agreed to participate in daily reflection discussion groups during the duration of the 2-week workshop. This group was a separate sample of youth from those who participated in the CAB focus group discussions, with the exception of 2 participants who were part of both the CAB and the films. Their consent process included an agreement for their likeness to appear in video and audio produced as part of the telenovelas. Each participant first engaged in an open-ended interview with the production team at Outside the Frame. This initial audio and video recorded interview was guided by the themes explored in the CAB focus groups (i.e., building solidarity based on intersectional marginalization, bullying, navigating social relationships and institutions, the meaning of citizenship and responsibility, and accessing health and social services). The goal was to identify stories that dialogued with the findings from the CAB focus groups based on the personal experiences of the film workshop participants. 

### 3.3. Data Analysis

Community-engaged data analysis included six Latinx youth and young adults (e.g., four college students who were 4-H alumni and two doctoral students with over 2 years of experience volunteering in 4-H as mentors). Engaging members of the community in the analysis process contributes to the rigor and trustworthiness of our interpretation [58]. Data analysts received CITI training and were added to the IRB protocols as study team members, and 3 young analysts were trained (NV, CDLT, IE) more extensively by the principal investigator (JG) in coding and memoing. Focus groups were transcribed verbatim in English and Spanish. Transcripts and other media files were uploaded into Dedoose for data management and analysis. Data were coded and analyzed by a research team using an immersion–crystallization approach [59] to describe salient patterns, compare narratives across stakeholder groups, and identify ways in which the intervention can generate critical consciousness. 

First, the team developed a priori codes from the theoretical framework and focus group guides. We met on a weekly basis to discuss and refine the initial organization of a start-list of codes into code families, to reconceptualize codes, and collapse redundant codes to ensure parsimony [60]. One team member was assigned with keeping a historical record of codebook changes. The codebook was structured by: code family, child code, detailed definition with inclusion and exclusion criteria, and an example of a phrase or quote from the data. 

Second, after developing the a priori codebook, each analyst read two transcripts in Microsoft Word, using comment boxes to apply a priori codes, and identified open codes not represented in the start list. Each member developed a list of emerging codes, which we compared at our weekly meeting to determine if the code could be captured by changing an existing code’s definition or whether it was conceptually different and needed to be added as a unique code or family. We discussed text segmentation and reached consensus about how much text should be selected when tagging narratives with codes (i.e., 50–200 words). The codebook was then edited by the coding leader to include new additions and reconceptualizations, collapse redundant codes for the second time, and describe the changes made in the document tracking historical changes to the codebook. Transcripts were subdivided among coders. Next, all coders applied the full codebook to 2 transcripts using Dedoose and met to discuss consistency in code application, refine definitions, and text segmentation. The coded documents were reviewed by the coding team leader to calculate interrater reliability [61], and this process was repeated until an interrater reliability of >0.80 was reached. 

Third, during the coding process, we developed memos to begin the process of interpretation [62]. The PI trained team members on the use of memos. The first memo was about each analyst’s subjectivity and perspective, including consideration of social identities (race, ethnicity, sexuality, participation in 4-H, religion, values, and SES). Subsequent memos dialogued with the theoretical framework (e.g., concepts of internalized stigma and marginalization, intersectionality, mutual identification, solidarity, and systems of oppression). Memos included data excerpts, preliminary interpretation, and analytic notes based on subjectivity. Memo writing and interpretation of memos were led by one 4-H alumnus (CDLT). She presented several analyses she compiled through memos to the larger group of 5 youth analysts for their critical reflection. Memos were linked to transcripts in Dedoose. 

In a parallel process, we coded participant notecard responses about factors that lead to social isolation and those that protected youth from or that addressed social isolation. First, an Excel worksheet was created noting participant demographics and the notecard responses. Next, we created 4 categories of codes to sort these responses: (1) Individual-level (traits/personal status, attitudes, proximal health risks); (2) Interpersonal (family, peers, mentors, other support systems); (3) Community/Cultural expectations and representation; and (4) Institutions (spaces, policies, laws, practices) and material resources. After each card response was categorized, we quantified the number of responses per level/category per individual. We calculated the proportion of responses per category for each CAB-type by dividing the number of cards in each category by the total number of card responses. In combination with the community-engaged memoing and critical reflection, this iterative process was used to identify salient patterns, emerging themes, representative quotes, and theoretical interpretation.

## 4. Results 

We structured our results by (1) Describing how stakeholders *reflected on and decoded* intersectional isolation on the individual, community, and structural levels; and (2) Identifying ways that stakeholders suggested *taking action* by improving access to resources to address social isolation and culturally competent healthcare as well as by co-creating an enabling social environment in 4-H.

### 4.1. Reflecting and Decoding 

Many of the individual-level perceived causes of social isolation that Latinx LGBTQ+ youth identified suggest that they may be internalizing intersectional stigma. They believed that attributes such as “being too dark”, “if you are ugly”, “not having social class”, and “not knowing English” caused social isolation (See Table 2). Latinx youth who only spoke English, or who were less comfortable speaking Spanish, experienced shame and isolation in Latinx communities and families. 

Several participants elaborated on how double-passing as White and “straight,” or as a cisgender woman was a privilege, at the same time that the ability to pass was a source of internal conflict.

*For a White LGBTQ person, they can hide their sexuality a little bit easier than a Latinx person who sometimes is not White passing and doesn’t have that privilege of hiding that part because you also have that other layer of not looking quite... So sometimes it’s a double whammy of sorts. There’s one community that’s lowkey racist towards you and has these assumptions of you: “Oh, you’re promiscuous, spicy [gagging], and like stuff like that.” But then you have…this other [Latinx] community on the extreme side of things think that you deserve to die for who you are. It’s going against God; it’s going against your family.*

(Latinx LGBTQ+ youth, CAB)

At the intersection of LGBTQ+ and Latinx communities, most LGBTQ+ youth experienced identity strain that left them feeling “alone”, “feeling like you’re not worthy because you don’t look in a certain way”, and lacking someone in whom to confide these feelings.

*Kind of confronting your own identity can be really hard when you’re not surrounded by people like you…thinking of examples in middle school. I’m White passing. When people say things around me that are really offensive like about Mexicans… They be like, “Those groups of girls are so annoying. Those loud assed Mexicans...” “You know I’m Mexican, right?” But they are like, “You are not THAT type of Mexican.” Like that stuff it puts you in a place where you are, “What type of Mexican am I?” You know like that is a cause of social isolation because I thought these people were my friends, but they are completely rejecting a part of my identity. Should I reject that part of my identity? …I don’t who do talk to about this because like I’m 12. I didn’t talk to my parents about this stuff, you know? It’s just you don’t know how to feel, and you don’t know who to talk to. Because…What do I do? Who am I? What am I? It’s so weird… The word Chicana wasn’t even like never heard of it until I was 19. Just being able to be honest about your own identity and have people accept it. There’re so many different levels to it. There’s a group of people who like maybe might be comfortable with my sexual orientation, but are they comfortable with my race and ethnicity? It’s just so many yeah, it’s a lot.*

(Latinx LGBTQ+ youth, CAB)

Beyond having to negotiate their racial/ethnic and LGBTQ+ identities in social relationships and within themselves, these youth were challenged by colorism. The social hierarchy of skin tones made both “White passing” and Afro-Latinx youth feel like they did not belong in their own families and among their racial-ethnic peers. They identified several ways in which community norms and cultural representations generated internal conflicts as depicted in Table 2.

*I don’t feel accepted in the Latin community nor the Black community nor the LGBTQ community. Ever since I was around 12, I knew I was attracted to both male and female but my family frowned upon it. In my family, they train girls at an early age to be a house wife, by buying easy bake ovens, dresses, and bows. I would go to church and the preacher said “God made Adam and Eve not Adam and Steve…I consider myself Afro-Latina because my mom is Dominican but people in America not familiar with the term. Like if I say something in Spanish, Black people would say “you’re black, you only speak English,” like there isn’t black people all around the world. And I asked one of my friends should I join Latino Leadership. He said, “No,” because I wasn’t light enough, my Spanglish wasn’t good enough, and I somewhat believe him. I hate that my family stripped out culture to fit in America. But Tuesday I’m joining Latino Leadership. I don’t care if I’m too dark.*

(Latinx LGBTQ+ youth, CAB)

This participant was struggling to participate in a Latino Leadership group because she feared that her skin color and language abilities would lead to her social exclusion. Several LGBTQ+ CAB participants identified having mentors and becoming leaders as a strategy to address social isolation, and “defend others from being attacked, treated badly, and from being badly talked about.” CAB members who had suffered social isolation saw themselves as well-equipped to help others based on their personal experiences. Some suggested “breaking expectations” and representations in the media to “show stories that subvert stereotypes or shift them from general to specific”, and to “debunk myths that Latinx communities have about LGBTQ identities.”

As shown in Table 3, according to 4-H Latinx youth, most of the factors (60.2%) that led to social isolation among their LGBTQ+ Latinx peers were at the individual level, including their status as LGBTQ+, “not identifying with any sex”, “being a minority”, “being disabled”, “introversion”, “competence”, “inability to communicate”, and others included in Table 3. Moreover, 4-H alumni mentioned “being homeless” as a source of social isolation, but they did not initially identify the legal structures that systemically exclude Latinx communities from the labor force or from stable housing as a part of the problem. Youth who listed “being undocumented” did not identify the need to change laws that place undocumented youth in situations of vulnerability. The implication of placing the responsibility for social isolation on the individual suggests that by simply “being” a certain way (e.g., darker skin, trans, or undocumented) youth are somehow responsible for their risk of social isolation.

Similarly, parents identified many individual traits (e.g., “color of skin”, “lack of identity”, and “feeling inferior because of language, race, color, sexual preference”) that result in social isolation and frequently used the word “normal” to describe heterosexual, cisgender youth, and “sexual preference or tendency” to describe sexual orientation. Underlying this framing was implicit bias—the assumption that LGBTQ+ youth were social deviants and that their current situation was a social illness. In fact, several of them believed that youth became LGBTQ+ because of childhood trauma, such as “suffering sexual abuse which leads them to decide not to have relations with the opposite sex and not have trust to tell someone about it.” The majority (51.6%) of the explanations parents gave for social isolation were at the interpersonal level, as shown in Table 4, indicating that children were socialized into isolation due to communication problems between parents and children, mothers not wanting to contradict the father when they inculcated masculine norms, as well as avoiding “getting close to other social groups because of not having a legal situation in a foreign country.” Parents also conflated sexuality and gender identity in statements such as, “he is gay, but he doesn’t dress like a woman.” 

Parents identified ways in which their experiences with marginalization due to nationality, language ability, color, and poverty allowed them to connect with the social exclusion that LGBTQ+ youth experienced. For instance, the struggle as an undocumented immigrant to receive social services without “papers” can be likened to those trans people face when they cannot receive gender-affirming care due to their gender documentation. Reflecting on the experience of shame among Latinx LGBTQ+ youth, parents drew parallels with the cultural shame they observed in their children about their Mexican identity.

*Our children suffer so much because they lack identity. One of my daughters told me, “Look, I don’t feel like I’m Mexican, and I’m not American.” I was not ready for that and didn’t know how to respond. So, I asked, “Why do you feel that way?” [She said,] “I know that I’m Mexican because you and dad are Mexicans. But I wasn’t born in Mexico. I was not raised in Mexico, and I don’t know anything about Mexico. I’m not American because I’m in the middle of all of the White people and I’m not like them.” …God illuminate and help me! … because kids suffer and they keep many things in silence. That’s how [LGBTQ+] people suffer rejection since their childhood, traumas from different situations… for the gay it’s triple, it’s like seeing him as abnormal. And on top of that, there is something extra, right? So, I told my daughter, “It’s true that you didn’t grow up in Mexico and you don’t know anything about Mexico, but don’t feel that way. It’s good that you are looking at that, that you are asking those questions. Maybe what you need is to get more involved in the Hispanic community and with young girls here. Because remember that here in this country we are a mix of cultures.*

(Latinx Parent, CAB)

Despite their difficulties understanding their children’s differences in gender and sexual identity, these narratives establish a foundation of willingness to engage in critical consciousness-raising in which parents and 4-H alum identified how their personal experiences with social isolation allowed them to empathize with LGBTQ+ youth. This was the basis for solidarity: parents recognized that by allowing a context where discrimination can occur against some youth (e.g., LGBTQ+ youth) they were contributing to a system of oppression where their children were challenged to negotiate rather than celebrate all aspects of their identities. 

### 4.2. Taking Action to Address Social Isolation in 4-H, Broader Community, and Healthcare Settings

#### 4.2.1. Within 4-H Youth Development Programs

Our multi-stakeholder analyses allowed us to identify ways in which we could modify the social environment in 4-H by building a mutually supportive environment for positive youth development and critical consciousness. To achieve the second objective of our analyses, we describe the perceptions of Latinx 4-H alumni and 4-H Latinx parents. 4-H alumni engaged in authentic dialogue in our community-engaged data analyses, identifying how language can generate the shame and blame that isolates LGBTQ+ youth. In the following analytic memo, a cisgender female, Mexican 4-H alum critically reflects:

*As I come from a small White conservative city, I think the mere act of touch or looking for too long was considered “gay.” I think this is terrifying for gay and queer men, too, since it’s difficult to make friendships with other men. I’m thinking back to the transcript with the LGBTQ+ CAB who mentioned that queer men are in need of emotional spaces, friendships, platonic intimacy since they often have access or platforms available for romantic relationships but not for the latter. So, yes homophobia impacts us all. It doesn’t allow for platonic intimacy or the building of relationships, especially with men, and I’m thinking it’s even more critical with men of color. Also, gay being used as an alternative word for “messed up,” or “weird,” or “strange” is something I definitely still hear at times. I think interrupting those comments, especially as leaders in the community or as staff in 4-H is super critical, because it would ideally try to help youth question why they attach negative associations to the word “gay” and also that being “gay” is fine and it’s a valid identity and just because you don’t identify as such doesn’t mean it’s not valid, normal, and worthy of respect.*

Moreover, parents shared personal experiences as chaperones in 4-H that acknowledged how social isolation placed LGBTQ+ youth in danger of suicide and self-harm.

*Sometimes the result is suicide, with people who cannot accept themselves, or maybe they’re afraid. In the 4-H camp, I met a child. I spoke with him in the last 4-H camp. He told me he never left his room. He was very quiet. His room was very dark, and he wore all black. He said, “Because I don’t want to leave the room.” He doesn’t want to accept himself; he is gay. His parents told him, “You don’t you have a girlfriend?” He’s very scared. I told him the story of another child, and that there was nothing wrong with being the way he was. I tried to help him. I told him we are all human beings deserving the same respect. He told me his parents asked him if he was gay, that he said, “No, no, no!” He wants to try to become a leader in the next camp, and I told him to try hard (“hechale ganas”). I think that’s why they commit suicide, because they don’t want to accept themselves due to fear… and sometimes they harm themselves.*

(Latinx Parent, CAB)

Like the LGBTQ+ youth and 4-H alumni, 4-H parents encouraged youth to participate in leadership activities to develop a sense of belonging. Although parents’ narratives revealed implicit biases, they also suggest a willingness to tap into their “compassion” to address isolation.

*Teachers have a lot to do with it [social isolation] because they don’t pay attention to students in case there is bullying against a gay or any other person. Teachers need to do a better job of observing young people…and of explaining that if they feel threatened by something, they can trust them and nothing bad will happen. I heard about a mom that felt she had to send her kid back to Mexico because he was bullied for not being born here…So… imagine those who are gay, and that are not heard in schools. If they go to their parents, their parents are working and little involved, that’s why they go as far as committing suicide, because they are not heard by each other or by anyone else, they are isolated.*

(Latinx Parent, CAB)

4-H alumni in community-engaged data analysis were able to go even further to acknowledge that “White supremacy” and other system oppressions (“socio-economic status stereotypes”) were underlying sources of social isolation for both LGBTQ+ and Latinx people of color. This is exemplified in this analytic memo by a 4-H alum:

*Stereotypes are perpetuated by people in power and people who benefit from these stories often even Latinx people, too. It deliberately erases people’s unique lived experiences---but more so Black Latinx, Afro Latinx, Afro-Mexican, Afro-Indigenous, and Indigenous people of Latin America… At the 4-H summer camps there is hesitancy to discuss how Latinx youth can perpetuate racism… I know one youth came up to me and said “but, I don’t get it…” and “also, why is this relevant/necessary---racism doesn’t hurt me” This youth was not only white passing but also identified/most likely still identifies as a cisgender heterosexual young Latinx man… We had Indigenous youth from Oaxaca, MX whose Spanish was their second language and they were learning English. We had immigrant Black Colombian youth who had recently immigrated to Salem, Oregon and who didn’t speak English to the level that they felt comfortable with… There were definitely moments that I witnessed othering and tried to find ways to talk about it--but it was challenging. The dominant group was definitely migrant Mexican-American youth... How do we unpack that? How do we bring in stories? How do we talk about power and privilege--how do we help frame privilege as a responsibility to reflect, to give space, to speak up if needed? In light of what is a big conversation around police brutality and Black Lives Matter, Latinx people are not exempt from these topics. We need to talk about anti-Blackness and the contributions/the deep roots of Africa in Latin America. The story of who we are involves all of us--colonization and slavery still impact our lives--past and present.*

#### 4.2.2. Within Broader Community and Healthcare Settings

##### Safe and Brave Community Spaces

Considering broader structures beyond 4-H, LGBTQ+ youth offered a wide variety of structural changes that could reduce or prevent social isolation in their communities (See Table 2). Most of their suggestions constituted creating safe and brave spaces in schools, churches, communities, and healthcare settings that were inclusive of youth with intersectional LGBTQ+ Latinx identities.

*Living in a more rural area of Oregon, a predominantly White state, there are few spaces for Latinos to gather socially and even fewer for LGBTQ people. Spaces for Latinx LGBTQ people are almost nonexistent. Latinx events are sometimes homophobic and LGBTQ events are always predominantly White and male. This can make it hard to find peers with similar identities. In my experience, it’s often difficult for LGBTQ Latinx to find acceptance from their families and Latino communities because of stricter cultural norms around gender and sexuality as well as homophobia from religion. I have also found that Latinx identity is not always celebrated or acknowledged in predominantly White academic environments. LGBTQ events for younger groups as well as Latinx cultural celebrations and groups such as a GSA (gay straight alliance) and ALAS (Latin American student groups) gave me and others a place to meet and create relationships with other Latinx LGBTQ groups.*

(Latinx LGBTQ+ youth, CAB)

Another participant noted, “Without a gay bar or a teen center focused on gay youth —which these places do not exist in rural communities—they have nowhere to go to find people or build a social network.” 

##### Affirming Sexuality Education

In addition to brave community spaces, LGBTQ+ youth reported needing access to gender-affirming and culturally appropriate sexuality education that went beyond the focus on HIV and AIDS.

*When you’re in high school and middle school you only learn about straight sex and that closes so many doors already in terms of everything, everything! Growing up gay and not even knowing what gay stands for or what sex is... You just don’t get the same treatment obviously as everyone else… Shows you like this kind of space isn’t for you. Like this curriculum isn’t for you. You have to learn this stuff on your own. You have to go online. You have to ask somebody. But as kids you aren’t going to ask your parents; chances are, you are not out to your parents, maybe you don’t even know your identity. You just know I don’t like the opposite gender or whatever gender. It makes kids feel like isolated. Just isolated in another way. I don’t get to learn about my life, my risks, and any of that stuff.*

(Latinx LGBTQ+ Youth, CAB)

This narrative excerpt reveals how not including diverse sexualities and gender in the curriculum led youth to feel isolated, unrepresented, and without any resources to learn about sexual health other than the Internet. Participants responded by claiming, “There’s not a lot of resources that can cater to that, unless it’s like towards those with HIV and AIDS”, and they felt that parents sometimes “disguised” their rejection with “concerned for your safety and health”, especially about the risk for HIV and AIDS.

*Latino communities can also not be so accepting of LGBTQ+ Latinx folks, because especially in families, it’s disguised as concerned for your safety and health, and all these other things, which is also a messy thing. It’s fair to be concerned for your loved one and their safety. Know the world is not super accepting of them. But it’s also like kind of controlling. I don’t want you to be who you are because I want you to be happy, I don’t want you getting hurt. That sort of thing…like you can make it try to come off of good intentions but once you get down to the nitty-gritty or down to take off all the layers they can be filled with negative intentions and impact.*

(Latinx LGBTQ+ Youth, CAB)

LGBTQ+ youth revealed the need for sexual health services that engage with the diversity of sexualities and genders, which includes mental and physical health issues that affect LGBTQ+ youth disproportionately (e.g., suicide, self-harm), and that teaches them about the history and current reality of the HIV and AIDS pandemic. Youth wanted more consideration of LGBTQ+ issues in the school curriculum, such as LGBTQ+ history, to feel represented and included in meaningful ways. 

##### Mental Health Services

Several LGBTQ+ youth noted that mental health services were particularly unavailable in rural parts of Oregon and even in larger towns such as Corvallis, especially for a “transgender person [who] can’t just go anywhere [for healthcare].”

*I think it maybe you have or get to the point to where you think you have the resources to find a therapist or a counselor. Like we’ve already talked about the increased mental issues with LGBTQ. If we are looking at how to solve that… we need more providers with experience. We have a lack of mental health providers in general and then we get to ones who actually who have actually worked with LGBTQ people or youth, and that is very few, especially in the context of rural. Even more so, I tried looking to see how many providers in Corvallis have experience working with a transgender person, and I found one... That is obviously a problem.*

(Latinx LGBTQ+ Youth, CAB)

Both LGBTQ+ youth and health providers identified the most socially isolated group to be Latinx transgender youth who were rejected by their families, who subsequently became homeless, and lacked access to social support and gender-affirming healthcare. A trans participant recalled being forced to come out and being rejected by her family when she began visiting doctors for gender-affirming hormone therapy:

*There’s a lot of pressure to be macho that I experienced, and I felt a sort of resentment for not living up to that, experiencing the intersectionality of both racism and homophobia/transphobia. I experience a good bit of homophobia from, as I viewed it, religious influence, particularly Catholicism, which was the main religion in my family. To give a direct anecdote from my own experiences, I hid the fact that I was transitioning from my family for a long time. The start of that process involves a lot of appointments, which my mom took note of. I was heading to see a doctor for hormone therapy, and my mom cornered me and asked about the frequent doctors’ visits. The idea of coming out was extremely uncomfortable. I continued to hide my identity from friends and family out of fear. I eventually came out, but became homeless not long after because of it.*

(Transfemme, 22-year-old)

Thus, access to gender-affirming and mental health services was challenged by an unsupportive family. In the following excerpt, a 47-year-old Latinx healthcare and social services provider shared a story of how access to mental health services was shaped by the family’s religious norms.

*A 16-year-old, bisexual femme identified youth was afraid to access mental health services due to her mother being religious— forcing her to “come out” to access a counselor or explaining the need to her parents. In addition, the stigma of going to counseling in the Latinx community. Last time this youth tried to talk to her mom about being bisexual, she told her to wait until she was 18. This youth overheard her brother and mom talking about being gay being a sin. Youth attempted to run away, and her parent overheard her telling the cops, “They won’t accept me because of my sexuality.” The police officer comforted the youth due to the cop also being LGBT and understanding. This youth feels like she should just put off going to counseling and queer groups until she goes to college and moves out. Her older sister who also identifies on the queer spectrum reached out to provide support. But her mother took her phone away for the time being limiting her outings. The youth did go to her school counselor and her older sister helped her make a safety plan, but because she is underage there has been limitations in interactions.*

Many participants shared stories that confirmed the role of family and religious rejection in resulting isolation and homelessness, as well as the institutional reasons that have left youth with a sense of not belonging in homeless shelters and community health clinics, as described in the following section.

##### Institutional Culture in Healthcare

Healthcare and social service providers identified sources of structural social isolation, primarily those concerned with community and cultural representation (30.2%) and institutions/policies (28.1%) as shown in Table 5. 

Even within institutions that have official policies meant to be inclusive, social exclusion challenged the lived experience of their clients. A 31-year-old community mental health worker told the story of:

*A trans Latina woman accessing services in the homeless youth continuum (HYC)…regularly struggles to get her needs met, and is often excluded from services due to various behaviors: staying past closing in shelter, taking “too long” in day-program showers, making sexually explicit comments to staff. Often, she is late due to needing more time to shave, do her hair, apply make-up, etc., so that she can present in a way that feels safe. Additionally, few staff speak Spanish, so she struggles to be understood or connect with staff…*

Several providers spoke about Latinx LGBTQ+ youth encountering nuanced language barriers, both because they spoke limited English and because of the gendered nature of the Spanish language. The importance of language was most pronounced when staff and volunteers misgendered youth in drop-in services at community health clinics. A 25-year-old, nonbinary, White-identified youth specialist explains this dynamic in their experience:

*I coordinate all of the volunteers at the [community health clinic for the homeless] kitchen… And volunteers don’t have tons of training with this sort of thing…sometimes don’t understand stuff, especially when it has to do with race stuff, or LGBT stuff or both. And, um, one of the clients that we have is trans [pause] and doesn’t appear to be trans... And so, he just gets misgendered constantly by staff who don’t know better yet…He said that the staff keep misgendering him all of the time because of both the race barrier and the cis-trans barrier, that it’s just not worth it to try and say anything… I bounced around to the volunteers that were working there, and said, “Hey that person over there, not a girl, don’t say things like that to him, he doesn’t like it.” …I’ve had that talk about 100,000 times at this job.*

Staff and volunteer turnover challenged their ability to create an inclusive space for their youth. For Latinx trans and non-binary youth, the need to show legal documentation during case management constituted an institutional form of isolation exacerbated by “fear and anxiety”. According to a 27-year-old cisgender, Mexican American case manager,

*Applying for Oregon Health Plan (OHP, Oregon’s Medicaid program) benefits, youth are not able to understand legal documentation’s language. Not being aware of their legal status, and also not fully understanding other names documentations are called. I’ve noticed asking for any legal documentation scares most of my clients away. Then this fear or anxiety prevents them from accessing benefits/resources. OHP person is not always aware of how triggering a simple question is. In this situation, the client never reached out to me. The benefits specialist did. I am currently navigating the communication between both parties, but many people do not have case managers.*

Table 5 presents the variety of factors providers identified to address or prevent social isolation at the level of creating institutional safe spaces, inclusive policies, and providing equitable access to material resources. These included: “Trauma-informed paperwork”, “Educating benefit specialists how documentation requests can be triggering”, “Explain what IDs are used for and that they will never disclose documentation status to anyone”, and consistent training for all staff who are interacting with youth. In sum, both LGBTQ+ youth and providers emphasized that in order to address social isolation, youth needed an enabling social environment that fostered positive identity development, supportive relationships, and access to health and social services. 

Our results suggest critical consciousness is needed to shift the responsibility to the community norms that perpetuate negative representations of LGBTQ+ and Latinx youth of color and to the lack of institutional practices that enable positive social connectedness among them. This distinction highlights the importance of shifting the framing of social isolation as a problem where LGBTQ+ youth have personal characteristics and attributes that put them at risk to an understanding of the contextual, institutional, and system factors that place these youth in vulnerable circumstances. 

## 5. Discussion

### 5.1. Reflection and Decoding the Problem

Our study indicates the importance of framing social isolation as the product of oppressive social and institutional environments, rather than as an individual failing. We draw a critical link between social inclusion in positive youth development programs such as 4-H and the overall health and well-being of LGBTQ+ Latinx youth. Our analysis uniquely engages the perspectives of various social stakeholders to modify the social environment for youth who are marginalized due to their race/ethnicity, sexual orientation, gender identity, and geographic marginalization. 

Latinx LGBTQ+ youth identified various individual-level traits they attributed to their experiences of social isolation (“being too dark”, “if you are obese”, “self-pity”, and “not worthy”), which indicated they blamed themselves and internalized their oppression. However, they identified a wide variety of cultural and institutional drivers of social isolation that could be modified through tailored interventions. Similar to Latinx LGBTQ+ youth, 4-H Latinx youth primarily perceived social isolation as attributed to individual-level factors (e.g., gender identity and minority status), whereas 4-H parents primarily described both individual- and interpersonal-level factors driving social isolation (e.g., childhood trauma from family and religion, sexual trauma, poor communication with parents, and hesitancy to get close to other social groups). LGBTQ+ service providers acknowledged the importance of incorporating “trauma-informed care” practices in clinical, organizational, and institutional spaces to mitigate marginalization in LGBTQ+ youth’s daily lives. By categorizing and decoding the meanings of social isolation from various stakeholders, we provided a multidimensional perspective, which is necessary for devising a solidarity-based solution to address intersectional social isolation in 4-H. 

Existing research on the broader health implications of social isolation among LGBTQ+ youth is highly focused on individual-level exposures and psychosocial outcomes. Individual level exposures include perceived loneliness and burdensomeness, a sense of belonging and connectedness, expected rejection, a sense of victimization, and internalized intersectional stigma [1]. These individual perceptions resulting from social experiences lead to individual health outcomes such as psychological distress, depression, self-harm, suicidal ideation, suicide attempts, substance use, and sexual risk taking [1]. This can hinder LGBTQ+ youth involvement in social and political processes in which they can advocate for enabling environments that allow the LGBTQ+ community to thrive [1]. Interventions that increase social connectedness and well-being are needed to reduce negative health outcomes and increase political and civic responsibility and participation [63]. There are few studies on programs or interventions addressing social isolation from an intersectional lens [1,22,23,34,64].

In addition to the critical consciousness approach, our analysis employed the constructs of intersectional identities and internalized stigma. Theories of intersectionality draw attention to the multiplicative social strain experienced by individuals who hold several marginalized identities [65,66]. The internalization of stigma describes a process by which individuals reproduce the stigma inflicted by systems of oppression, thereby incorporating learned stigma in their self-concept [67]. As a result of internalized intersectional stigma, youth in our study experienced identity strain and difficulty in managing their diverse social identities across various social situations. Youth used visibility management—the selective expression of one’s identity [1,5,49]—to navigate social environments that negated one or more of their social identities. They intentionally made themselves more or less visible to avoid a perceived threat in their environment. Prior research on visibility management describes this selective identity expression as a strategy to navigate social contexts perceived to be unsafe [1,5,49]. However, the differential privilege of having access to visibility management as a potential strategy reinforces social hierarchies set up by systems of oppression as some aspects of identity, such as shades of skin color, strengths accents, and degree of language fluency, are variably visible.

In our study, visibility management strategies often led youth to feeling like they “lack identity” or a sense of self, “not worth it”, and “alone.” Participants in this study expressed frustration, or identity strain, in having to maintain vigilance over the visibility of their identities. This observation confirms previous findings on the social strain LGBTQ+ youth experience during childhood development [5,22,49,68]. However, prior studies have not generally considered the compounding effects of managing the visibility of multiple provisional identities, and their relationship to social isolation and stress [5,22]. Youth in our study found it difficult to navigate social environments in which peers accepted one aspect of their identity (e.g., Latinx) but not the other (e.g., LGBTQ+), resulting in the intersectional isolation from ethnic enclaves and White queer spaces. Participants were able to critically reflect on how those spaces shaped their need to manage identity visibility. Prior studies have found lateral oppression (i.e., within-group microaggressions and marginalization) provoked a stronger physiological response (e.g., higher cortisol levels, higher heart rate), an emotional response (e.g., anger), higher risk-taking behaviors, and violence [69,70,71]. For this reason, it is necessary to create safe spaces to engage in authentic dialogue about power and privilege within members of the same identity groups (e.g., Latinx, LGBTQ+, etc.) and unpack how marginalization is perpetuated within identity groups.

Parents in our study recognized a parallel between their own experiences of marginalization due to nationality, race, color, and poverty with the marginalization experienced by Latinx LGBTQ+ youth. A critical consciousness framework allowed them to engage in meaningful dialogue in which they can recognize how they play the role of both the oppressed and of the oppressor. For instance, parents reflected on challenges youth faced in affirming their Latinx identities, as their children struggled to navigate their Latin American and US nationalities. Participants drew parallels between the experience of “passing as White” (non-Latino) with that of “passing as straight”. The identity strain they observed in their own children and in other 4-H youth they chaperoned revealed that the consequences of social exclusion included suicidal ideation, which they agreed no child should experience. Our 4-H youth and alumni focus groups found Latinx parents’ coded homophobia was sometimes *disguised as concern* for HIV infection, pathologizing their children’s sexuality, instilling fear of violence and discrimination from society, and minimizing asexuality and bisexuality as a phase. This suggests that critical consciousness-based interventions are needed to help parents identify their coded homophobia and microaggressions that contribute to their children’s sense of isolation. 

### 5.2. Taking Action

Through a critical consciousness framework, participants were able to reflect on how institutional and social barriers to services and programs led to social isolation, and ultimately propose strategies for affirming the social wellbeing and health of Latinx LGBTQ+ youth. A critical consciousness perspective underscores the need to identify and modify social and institutional environments to address social isolation. 

#### 5.2.1. 4-H Youth Development Programs

First, our study highlights the need for interventions based on positive youth development (PYD) to engage youth in their communities, strengthen internal and external assets, and foster positive identity, relationships, and supports [72]. The 4-H program has been highlighted as a model for PYD programs. Through their longitudinal study, they have found their youth program participants are four times more likely to make contributions to their community, two times more likely to be civically engaged, and twice as likely to make healthier choices [73]. The Oregon 4-H Latino Outreach Project was developed to reach and engage Latino youth and families who may not feel welcomed in traditional 4-H settings [74]. The Latino Outreach Project has sought to “respect and reinforce the cultural identity of youth” by incorporating activities that “broaden cultural awareness and pride” [74] (p.4). In our study, we found that it was important to generate pride in Latinx and LGBTQ+ youth who felt like they did not belong in the communities they lived in. Like our community conversations, authentic dialogue is needed for people to examine their lived realities and explore ways their intersectional identities can be both oppressed and an oppressor. It is a way to openly acknowledge power imbalances that are socially corrosive. Rather than reinforcing dichotomies (e.g., LGBTQ+/cisgender heteronormative, White/Latino, or immigrant/US-born), a culture of authenticity is necessary to develop awareness, pride, and celebration of the self for those who endure systemic oppression through community and peer support. Our youth were interested in seeing more heroes, representing the hope that comes from finding a community of people who share a similar identity or lived experience. One youth shared, “We need a lot more of happy endings. We always see LGBTQ+ movies with someone dying at the end, there is no Cinderella happy ending.” This helps cultivate their ability to develop a positive, authentic sense of self. Incorporating pride and stories of hope through education that validates and celebrates intersectional LGBTQ+ lived experiences, emerges the importance of focusing on positive, enabling rights within organizations and communities that allow the individual to flourish.

In youth programs, there is an urgent need to move beyond just training staff in peer-to-peer anti-bullying practices. Staff should also be trained to recognize systemic oppression and be given the tools to actively improve the school’s institutional environment [75]. In the 4-H program, there is a need to add LGBTQ+ competencies within staff training [76]. 4-H staff in North Carolina reported low levels of knowledge or skills in how to serve transgender and gender non-binary youth or provide affirming resources for LGBTQ+ youth and families [76]. In addition, 50% of 4-H staff did not report using positive images of LGBTQ+ people in their programming, the majority did not share their own pronouns with clients (76%), and only 63% agreed that youth at 4-H events should be allowed to use a restroom that matches their gender identity [76]. This study also highlighted that 4-H rural professionals scored lower in LGBTQ+ knowledge, skills, and disposition towards LGBTQ+ training and practices. However, overall, staff demonstrated high levels of interest and an agreement that they needed training to better support LGBTQ+ youth. In addition to teachers and after-school providers, school-based health centers (SBHCs) can also provide culturally affirming care. However, a previous study looking at LGBTQ+ quality of care at SBHCs found that 54.5% SBHCs had no general staff training on providing care for LGBTQ+ youth, 23.5% included gender pronouns in intake forms, and 53% SBHCs reviewed materials for harmful LGBTQ+ stereotypes [77]. Walia et al. (2019) found that cultural competency training can be leveraged to improve LGBTQ+-specific cultural competency [78]. Training staff in schools and clinical health care settings is key to developing more intersectional LGBTQ+ advocates that demand policy changes within their organizations and from the political institutions influencing them.

#### 5.2.2. Safe and Brave Spaces

Second, participants expressed a need for identity-affirming and safe spaces in various community settings, such as schools, churches, and the broader community. Like existing research on the importance of allyship within safe spaces, youth in our study highlighted the importance of having mentors, leaders, and allies that support [22,23,68,79] and actively defended Latinx LGBTQ+ youth from discrimination and bullying [11]. Safe spaces identified were often only inclusive of one axis of social identity (i.e., Latinx, LGBTQ+, or rurality); however, few were affirming of youth embodying multiple marginalized social identities. This led some Latinx LGBTQ+ youth to refrain from engaging in existing safe spaces or default to using visibility management strategies. To avoid the strain of visibility management that deteriorates Latinx LGBTQ+ youth’s sense of self, our study suggests an approach that builds mutual identification based on experiences of marginalization to generate solidarity in *intersectional* identity safe spaces.

To build these intersectional safe spaces, participants expressed a need to debunk myths and stereotypes about LGBTQ+ and Latinx identities through inclusive, fun, and educational activities that teach youth how to have brave conversations about social identity and provide opportunities to dialogue with their peers. Prior studies called for community-level education and awareness of LGBTQ+ people to increase opportunities for social connection, dispel myths and misconceptions about LGBTQ+ and Latinx people, and foster allyship in the broader community [1,22,68]. Intersectional safe spaces provide opportunities to develop allyship based on solidarity [80]. Genders & Sexualities Alliances (GSAs), which was originally named the Gay–Straight Alliance Network, were designed as student-run organizations and safe spaces for LGBTQ+ youth in middle/high schools. GSAs have adopted an intersectional framework, and recognizing the centrality of race as well as sexual and gender fluidity, they have now “emerged as vehicles for deep social change related to racial, gender, and educational justice” [81] (GSA, 2022) (Para 1). Providing opportunities for youth peers to connect, share their experiences, and affirm each other’s identities leads to advocacy and collective action [6]. LGBTQ+ youth organizations provide positive environments, relationships, and experiences and serve as vehicles for positive youth development. When these organizations additionally focus on serving youth of color, these spaces counteract the negative effects of internalizing their oppression by serving as an additional home and family where youth learn to cope, support others, and not only resist oppressive systems but challenge those ideologies [22]. These effects could also be created by intersectional pride-based events (parades, drag shows, balls, kiki lounges, etc.) and advocacy events for intersectional identities (Black Trans Lives Matter, Undocuqueer, Rainbow Caravans, etc.).

Safe spaces for Latinx LGBTQ+ youth seemed to be less available in rural communities, which reflects findings from previous studies on non-metropolitan communities where gender and sexual minority youth expressed difficulty in finding identity-affirming spaces and services [68]. Participants in our study from rural areas stated that “without a gay bar or a teen center focused on gay youth…they have nowhere to go to find people or build a social network.” The absence of these spaces in communities reduces the opportunity for socialization, impairing the development of LGBTQ+ youth and increasing social isolation [22]. This highlights the need to push for intersectional safe spaces in schools and rural communities to mitigate the negative health effects of oppression, build their peer network, promote positive identity development, and encourage youth and allies to push for systemic changes where they learn positive representations of LGBTQ+ people of color.

#### 5.2.3. Affirming Sexuality Education

Third, sexual health education programs, which are commonly nested within schools and other community settings, provide an important opportunity to address social isolation by generating intersectional safe spaces. Participants in our study acknowledged that parents and members of the Latinx community who opposed LGBTQ+ human rights target inclusive sexual education in schools. Although prior studies have applied a critical consciousness approach to sexual health among the LGBTQ+ community [49,79], there are no studies on how critical consciousness interventions can be used to address the intersectional marginalized experienced by Latinx LGBTQ+ youth. 

Our study suggests that Latinx LGBTQ+ youth need sexual health education that celebrates their diverse range of sexual orientations and gender identities. Our participants called for sexual health that does not reduce LGBTQ+ youth identity to simply, “at-risk for HIV and STIs”. Instead, they indicated the need to highlight social and institutional vulnerabilities and to recognize community assets that can help overcome these vulnerabilities. By building pride, youth sought to dismantle, rather than reinforce, stereotypes. Both the 2020 GLSEN study [11] and our focus groups emphasize the importance of including LGBTQ+ history in the curriculum. We go further to suggest the inclusion of an intersectional LGBTQ+ history that shows the importance of LGBTQ+ people across cultural contexts.

#### 5.2.4. Affirming Mental Health and Other Healthcare Contexts

Last, our study indicates the need for intersectional safe spaces within clinical settings to enable the availability of affirming physical and mental health services. Prior research indicates that support for identity-safe spaces is critical to addressing stigma, improving follow-up to care, skill-building, and hosting leisurely activities that welcome clients; although, this level of support is often not directly funded by program grants [82]. In our study, clinical spaces empowered youth by supporting their identities, for instance, when their parents wanted to “fix” their bisexuality or when they feared parents discovering that they are on gender-affirming hormone therapy. Clinical safe spaces play an even more important role in rural areas lacking a visible LGBTQ+ community of color. Our participants cited language barriers, fear of disclosing their legal status when asked to provide documentation, and fear of being perceived as a public charge as deterrents to engaging in health services. Intersectional safe spaces within clinical settings are necessary to counteract institutional oppression and help build trust in seeking primary and preventive health services among clients from historically marginalized communities (LGBTQ+, social class, immigration status, language, disability, etc.). 

For these reasons, providers and educators in our study agreed that specialized training was critically needed to sustain intersectional safe spaces within clinical and educational institutions. They described a need for trauma-informed care and the challenges of providing ongoing staff and volunteer training on affirming organizational practices. Participants highlighted a need to train their staff on how to affirm their patrons’ identities and avoid microaggressions, misgendering, and trauma-inducing language. Training staff was a challenge due to a reported high rate of turnover in community and health clinic-settings in which new staff are being constantly introduced. This problem highlights the need for training all staff in providing a welcoming environment through all the steps of the health navigation process, from inclusive intake forms and welcoming front desk reception staff, to referring folks to a team of providers who specialize in gender-affirming care for LGBTQ+ youth [83]. 

## 6. Conclusions

This study described a multi-stakeholder approach to building critical consciousness among Latinx LGBTQ+ youth, Latinx parents, 4-H youth, and providers servicing LGBTQ+ youth of color. This process highlighted the importance of tapping into core values and intersectional identities to build solidarity among and within marginalized groups to dismantle oppressive systems. Our work corroborates prior studies that indicate the importance of addressing the policy and legal landscape to create an inclusive environment to improve engagement in services and programs [1,22,68]. Addressing social isolation is critical in order for institutions to provide trauma-informed services for intersectional youth identities. At the same time, we need to celebrate intersectional identities within these institutional spaces through educational materials, diversity of staff, and identity-tailored programming for youth and allies. Funding for LGBTQ+ youth and ally programs that reflect on the intersectional social issues youth experience, engage peers in authentic dialogue, and move towards mutual identification, solidarity, and collective action are necessary for positive youth development and the health of all our communities.

## Figures and Tables

**Table 1:** Methods used, participant type, and domains.

Method	Participants	N	Main Domains
**Community Advisory Board Focus Groups**	Latinx LGBTQ+ youth, 18–24 years old	12	Bullying and resources to address intersectional marginalization; Factors that lead to and protect from social isolation (notecards)
	4-H Latinx youth, 18–24 years old	12	Bullying and resources to address intersectional marginalization; Factors that lead to and protect from social isolation (notecards)
	4-H Latinx parents	8	Intersectional marginalization; Meaning of citizenship; Community building to address social isolation; Factors that lead to and protect from social isolation (notecards)
	Health/social service providers	10	Resources necessary to address social isolation; Daily institutional practices in their workplace; Factors that lead to and protect from social isolation (notecards)
**Interviews with film workshop participants**	Latinx LGBTQ+ youth, 18–24 years old	9 (of 10 agreed to be interviewed)	Connect lived experience with themes that emerged in focus groups; Reflecting on Bullying; Civic Responsibility; Community building; Mutual Identification/Solidarity

**Table 2:** Latinx LGBTQ+ Youth’s Perspectives on Social Isolation.

	Leads to Isolation (Total Identified = 118)	Protects from Isolation (Total Identified = 118)
**Individual** Personal traits	*n* = 32 (29.4%) Examples: “Being too dark”; “If you are short”; “If you are ugly”; “Not knowing English”; “Not having social class”	*n* = 22 (24.4%) Examples: “Dye your hair”; “Able to socialize”; “Adventurous”; “Show yourself as strong and wise”
Attitudes	“Mental fear”; “Feeling like you’re not worthy because you don’t look in a certain way”; “Reduced knowledge of other and different communities”; “Reduced knowledge of self”	“Make out in front of them”; “Acceptance”; “Respect yourself”; “Say no to silence”; “Ignore mediocre people’s opinions”; “Being more open and vocal about these issues”; “Try to remove any bias or stigma from your mind”; “Knowing how to communicate”; “Trust in yourself and have confidence”
Proximal health risks	“Developing a form of eating disorder and body dysphoria”; “Suicide or self-harm and drug use because of depression and anxiety and isolation”; “If you are obese”	“Stop being depressed”
**Interpersonal** Family	*n* = 31 (28.4%) Examples: “Rejection from families and community that involves verbal, physical, emotional abuse”; “Family status (single parent, both parents, step-parent)”; “Rejection around coming out”	*n* = 24 (26.7%) Examples:
Peers	“Persecution, bullying around gender presentation from family and peers, being forced to present differently that you feel comfortable”; “Weak relationships to family, friends, and significant others”	“Talking to a good gay friend”; “Defend others from being attacked, treated badly, and from being badly talked about”
Mentors		“Out lists, openly out LGBTQ mentors, teachers, and adults”; “Adults should set the standard for acceptance, respect, and inclusion of LGBTQ+ youth”
Other support system	“I felt alone when someone didn’t ask me to go to their advocacy group”; “I feel isolated when people don’t include me”; “Envy in the LGBTQ community and we don’t support each other”; “Your community, beating each other up”; “Unsafe because of all the violence that you see happening today”; “Moving a lot/readjusting socially”	“Having in-depth conversations with LGBTQ+ members to learn and grow from it”; “Checking up on people you may not even know or are friends with”; “Going to support groups” “Talking to your boss about your problems”; “Educate others about red flags, stigmas, sexuality, gender, etc.”
**Community/Cultural** Expectations	*n* = 30 (27.5%) Examples: “Invalidation of your identity because you’re not really queer or not enough queer”; “I feel targeted when people say racial and homophobic jokes and comments”; “God doesn’t like LGBT”; “Machismo”; “Tradition (following tradition in order to make family happy)”; “All women can be is married to a man”	*n* = 12 (13.3%) Examples: “Breaking Expectations”; “Faith leaders preaching love and respect towards LGBTQ communities”; “Promoting empathy”; “Dispose of the myth that queers end up poorly adjusted”; “Debunk myths that Latinx communities have about LGBTQ identities”
Representation	“Sexualized”; “Unique dating challenges in reference to being LGBTQ and the marriage gradient/homogamy”; “I feel socially isolated when people think I’m dumb just because I’m Mexican”; “Lack of Latinx representation”; “A lack of understanding of what being LGBTQ is like/lack of empathy”	“Increase representation in media in regards to Latinx LGBTQ+ identities”; “Latinx media with positive portrayals of LGBTQ people living happy lives”; “Show stories that subvert stereotypes or shift them from general to specific”; “Searching on Facebook for people like us”; “Going on Grindr you can find people like us”
**Institutions** Spaces	*n* = 16 (14.7%) Examples: “Military”; “Bathrooms”; “Schools”; “Small towns”; “Feeling dysphoric and unsafe in extremely gendered activities or in cis/straight spaces discomfort getting in the way of pursuing athletic, academic, extracurricular activities”	*n* = 32 (35.6%) Examples: “Queer prom, LGBTQ pride events, Latinx and other cultural diversity days that educate students and community about history and present day issues”; “Educate local Latinx churches and places of worship on how to be more LGBTQ+ friendly”; “Create supportive spaces for Latinx parents of LGBTQ+ identities (brown and black PFLAG)”
Policies, laws, & practices	“Dealing with language barriers and the expectation in America for non-English speakers to accommodate English speakers”; “I feel isolated when I don’t get the same opportunities as white people”; “Lack of sexual education and gender orientation and identity, education makes youth feel isolated and leave them to search for their own educational materials”	“Opportunities for teachers, counselors, families to come out and announce themselves as allies”; “Educate Latinx immigrant families about LGBTQ+ identities and how to support”; “Involve discussion about sexual/gender identity in health and sex education curriculum” “Sexual and gender identity competency/sensitivity training for teachers/administration”; “Increase the amount of Latinx therapists that are also LGBTQ+ friendly”
Material and financial resources	“Homelessness after coming out or being outed”; “Location on the socioeconomic map”; “I feel used and hurt when I get underpaid. I feel like it’s because of my race and sexuality”	

**Table 3:** Latinx 4-H youth’s Perspectives on Social Isolation.

	Leads to Isolation (Total Identified = 118)	Protects from Isolation (Total Identified = 118)
**Individual** Personal traits	*n* = 71 (60.2 %) Examples: “Not identifying with any sex”; “being a minority”; “low academic performance or class placement”; “being homeless”; “not speaking English”; “people skills”; “being undocumented ^1^” “nationality”; “Maturity”; “introversion”; “inability to communicate”	*n* = 20 (16.9%) Examples: “Being comfortable with self”; “ability to achieve goals”; “people skills”; “time management”
Attitudes		“Open-mindedness”; “outgoing mindset”; “positive self-image”; “sense of belonging”; “feeling safe and welcome”; “educating yourself”
Proximal health risks	“Depression”; “mental instability”; “being disabled”; “physical differences”	“Physically healthy”; “able body”
**Interpersonal** Family	*n* = 15 (12.7%) Examples: “Toxic home life”; “domestic violence“; “family rejection and trauma”; “divorced parents”; “family immigration problems”	*n* = 42 (35.6%) Examples: “Supportive family and friends”; “healthy home life”
Peers Mentors Other support system	“No support from friends”; “bullying”; “Lack mentors” “Living far from community”	“Mentors” “Counseling”; “outreach”; “support groups”; “Surrounded by people that care”; “part of a team”
**Community/Cultural** Expectations	*n* = 9 (7.6%) Examples: “Community discrimination”; “lack of community acceptance”; “Latino culture”; “stereotypes”; “Religion”; “non-Christian religion”	*n* = 14 (11.9%) Examples: “Supportive spirituality”
Representation		“Positive and supportive social media”; “spreading awareness”; “your identified group being more positively represented through media”
**Institutions** Spaces	*n* = 23 (19.5%) Examples: “Extracurricular activity space”; “sports teams”; “rural spaces”	*n* = 41(34.7%) Examples: “Safe neighborhoods”; “geographic access to parks and libraries”; “access to involvement in clubs and teams, inclusive events in schools and cities”
Policies, laws, and practices	“Political parties”	“Access to information considered taboo”; “systems that are inclusive, flexible, and equitable”; “stopping all sorts of segregation or labeling of groups, us vs. them”; “empowering organizations”; “access to healthcare”
Material and financial resources	“Lack of telecommunication”; “transportation”; “low family income”; “poverty”; “homelessness”; “non-traditional housing situations”; “live in area with low resource accessibility”	“Access to transportation and secure housing”

^1^ Note: whenever an attribute was described as a way of “being” (e.g., being Latino, being a minority, being homeless, being undocumented, being disabled), we coded it at individual level. When the issue was framed in terms of policies or laws (immigration laws, legal system) or as a system level issue about access, inequity, or non-inclusive space it was coded at the institutional or resource/structural level. This distinction is critical because it highlights the importance of shifting the framing of social isolation as a problem where LGBTQ+ youth have personal characteristics and attributes that put them *at risk* to an understanding of contextual, institutional and system factors that place these youth in *vulnerable* circumstances. It is important to highlight that youth who listed “being undocumented” did not identify the need to change laws that place undocumented youth in situations of vulnerability. Analytically, this indicates that critical consciousness is necessary to frame social isolation as the result of contextual factors and institutional policies rather than as an issue of personal responsibility. In addition, there is another opportunity to generate critical consciousness in the fact that race and ethnicity were listed as traits that constitute *barriers* to social isolation. If instead youth were able to generate *pride* from those identities, they would perceive them as facilitators and sources of solidarity.

**Table 4:** Latinx 4-H Parents’ Perspectives on Social Isolation.

	Leads to Isolation (Total Identified = 93)	Protects from Isolation (Total Identified = 48)
**Individual** Personal Traits	*n* = 23 (24.7%) Examples: “Color of skin”; “undocumented/migration status”; “not being academically prepared”; “timidity”; “lack of identity”; “weakness”	*n* = 1 (2.1%) Example:
Attitudes,	“Frustration”; “low self-esteem”; “insecurity about yourself”; “feeling inferior because of language race, color, sexual preference”; “feeling guilt”; “loneliness”	“Express your identity without fear and with freedom”
Proximal health risks	“Depression”; “having a disability;”	
**Interpersonal** Family	*n* = 48 (51.6%) Examples: “Latino fathers tend to motivate their sons to watch pornographic movies or magazine, especially with women with little clothes on, which in some cases makes them feel uncomfortable and also encourage them to drink alcohol”; “Being forced, more among the Latino boys, to have sex from a very young age with women to show other men in their family that they are men”; “Silence of the mother to avoid contradicting the father”	*n* = 20 (41.7%) Examples: “When family motivates youth by talking to them and helping them economically”; “As parents, talk to our children and explain differences in gender (lesbians and gays) and teach them to respect others and accept them”; “Trust between youth and their parents to tell them everything, good and bad”; “Parents educate children about differences, timidity, and physical conditions, and teach them to respect and help their peers”
Peers/mentors	“Rejection from family, school friends, coworkers, church, and society, teasing, bullying and violence due to race, color, or sexual preference or tendency”	“Good friends that encourage them to succeed”
Other support system	“Suffering sexual abuse which leads them to decide not to have relations with the opposite sex and not have trust to tell someone about it”; “Fear of speaking and getting close to other social groups because of not having a legal situation in a foreign country”; “Live far away from their community and family so they do not find out about their sexual preference”	“Teaching our children to share and help in programs to become friends with LGBT groups although they do not consider themselves to be LGBT”; “Read and learn about diseases that we imagine are contagious and teach our children to respect others and to be respected”; “supportive school counselors”
**Community/Cultural** Expectations	*n* = 14 (15.1%) Examples: “Racism”; “Hispanic culture”; “Machismo in Latino culture that does not accept the LGBT group” “Lack of values in society”; “Lack of tolerance in their social surroundings”; “With their sexual preferences they are committing a grave offense against God”; “Cultural differences, many times they don’t feel included in the culture of the United States”; “Rejection of a disease like AIDS”; “Religion and church is not tolerant and single them out as sinner and people who are condemned”	*n* = 11 (22.9%) Examples: “Values of love, compassion, and care for every human being”; “In your community to foment a culture of tolerance and diversity”; “That churches don’t foment a stereotype of man-woman and don’t blame the LGBTQ community”; “Teaching that it is not a defect, or error, or sin, or that because you feel that way you are a demon like they claim in some religions”; “Teaching our community to understand and listen to why we need to accept their way of being”; “Churches with spiritual retreats for youth”
Representation		“Culture of tolerance in the media”
**Institutions** Spaces	*n* = 8 (8.6%) Examples: “Politics of intolerance in the family, religion, school, work and government”	*n* = 16 (33.3%) Examples: “4H as a program helps youth a lot”; “Schools that develop and have a culture of tolerance”; “Places, workshops, or programs that explain different migratory situations”; “Lift their voices and organizations that are obtaining a space in society”
Policies, laws, and practices	“Discrimination for different color of skin”	“Workshops in schools to train children, families, and teachers to ‘accept’ and ‘not to judge’ LGBTQ youth”; “Support from representatives and congress people that support expressions of respect for tolerance toward those who are and think differently”; “That law enforcement does not repress them and act with intolerance for their identities”
Material and financial resources	“Poverty”; “The migratory situation of their parents can cause insecurity”	

**Table 5:** Health and Social Service Providers’ Perspectives on Social Isolation.

	Leads to Isolation (Total Identified = 96)	Protects from Isolation (Total Identified = 85)
**Individual** Personal traits	*n* = 22 (22.9%) Examples: “Being of mixed race/ethnicity”; “Pigment”; “Being trans vs. gay/lesbian/bi”; “Language—not all Latinx speak, read, write Spanish”; “Gender roles and expression” “Criminal record”	*n* = 3 (3.5%) Examples:
Attitudes	“Punishing/snapping at people who make honest mistakes”; “Shame with weaknesses”	“Being careful not to let your personal trauma override others’ joy”; “Open mind to view and affirm others experiences”
Proximal health risks	“Disability;” “Drug and alcohol abuse;” “HIV/AIDS status;” “Unaddressed depression;”	
**Interpersonal** Family	*n* = 18 (18.8%) Examples: “Homophobia/transphobia in family”; “Family members exposing you to toxic communities”; “Parents marital status (married to each other, divorced and remarried to someone that’s unaccepting)”; “Immigration of family”	*n* = 14 (16.5%) Examples: “Parents having same rules for your kids regardless of gender. Encourage their dream. Not defining career paths by gender”; “Latino educator/volunteer translators for parent support groups”
Peers	Dating, coming out, asking out, lateral oppression, my bisexual partner is transphobic”; “’The struggle’ I face—my peers don’t understand how stressed I am, they insist ‘we all have stress’”	“Create/host peer support groups”
Mentors		“Having mentors that look like you”; “Lived experience to make connections”
Other support system	“Lack of affinity groups/social supports”; “Prioritizing ‘not rocking the boat’ over actual support”; “Forcing youth to come out”; “Safety concerns with online dating”; “Suffering abuse (sexual, physical, emotional)”	“Stopping to take the time to actually interact with those we serve”; “Chosen family, acceptance, no judgement or questions only love and support” “Community outreach to the Latine community, creating relationships for the youth to come to us”; “Resources for youth’s support system (adults in their lives)”; “Spiritual support”
**Community/Cultural** Expectations	*n* = 29 (30.2%) Examples: “Volunteers and training them to be more judge-free, most come with preconceived notions”; “Mental health, cultural shame, and difficulty breaking those associations”; “Perceiving LGBTQ as a mental illness that can ‘be cured’ or that you out-grow”; “Latinx community not allowing you to bring your whole self and checking your queerness at the door”; “Racist rhetoric from LGBT community”; “The community not understanding homelessness and youth”; “Religious expectations (sin/going to hell)”; “Machismo/gender role expectations”; “Generation gap”	*n* = 20 (23.5%) Examples: “Uplifting youth, letting them know their voices have meaning without critique”; “Do not assume all identities are expressed the same way”; “We understand we are interreacting with youth who are good kids surviving bad situations”; “Community building, focus on what we have in common”; “A welcoming and affirming church, knowing someone at their church is available to listen”
Representation		“Celebrate their culture”; “Music/poetry/art that represents the population”; “Books, websites that reinforce identity”; “Put up visible signs of support (safe space, rainbow flags, photos of diverse couples/families)”; “Use affirming language”; “Use our own pronouns more often during introductions”; “Online multi-lingual targeted adverts for youth resources”; “Latinx centered community websites”
**Institutions** Spaces	*n* = 27 (28.1%) Examples: “Queer spaces being historically white and cis”; “Lack of safe spaces”; “Our drop-in (Thursday) is at a church, it might be isolating for some”; “Oregon is super white—staff are super white”; “Online exposure of youth identities and outdated websites”; “Approaching youth can be risky, we don’t want to out them by assuming”	*n* = 48 (56.5%) Examples: “Host PFLAG at Latinx community center”; “Creating a space that isn’t white/heteronormative (food choices, signage)”; “Offering closed spaces for people of color or Latinx folks”; “Creating a space where questions and trying/failing is encouraged compassionately in staff”
Policies, laws, and practices	“Resigning ourselves to high turnover staff not investing”; “Seeing staff/volunteers’ good intentions and providing no further training”; “Tokenization”; “Electronic medical records system doesn’t always have clear SOGI [sexual orientation and gender identity] info—preferred names only seen on some screens”; “Applying for anything in the system and needing to provide ID”;	“More Latinx folks in positions of power, representation matters”; “Including Latinx queer and trans people in regular programming”; “Use open ended questions (re: SOGI, dating, etc.)”; “Hire participants into supportive roles”; “Bilingual staff and volunteers”; “Educating benefit specialists how documentation requests can be triggering”; “Explain what IDs are used for and that they will never disclose documentation status to anyone” “Inclusive youth homeless shelters”; “Job opportunities and skills mentoring”; “Opportunities/resources that don’t require legal [documented] status”
Material and financial resources	“Lower incomes because of discrimination”; “Lack of services that affirm all of their identities”; “Homelessness”	

## Data Availability

The data set presented cannot be shared publicly due to ethical, legal or privacy issues and in accordance with the consent participants provided on the use of confidential data.
